# Metastatic melanoma to the ovary in pregnancy: A case report

**DOI:** 10.1016/j.gore.2021.100859

**Published:** 2021-09-11

**Authors:** D.S. Huang, R.B. Hegeman, M. Roy, T.M. Prout, K. Swartz, M. Olsen, S.L. Rose

**Affiliations:** a University of Wisconsin, Department of Obstetrics and Gynecology, Madison, WI, United States; b University of Wisconsin Division of Hematology, Medical Oncology and Palliative Care, Department of Medicine, Madison, WI, United States; c University of Wisconsin, Department of Pathology, Madison, WI, United States; d University of Wisconsin, Department of Radiology, Madison, WI, United States; e Department of Pathology, University of Wisconsin, Madison, WI, United States; f University of Wisconsin, Division of Pediatric Oncology, Department of Pediatrics, Madison, WI, United States; g University of Wisconsin Division of Gynecology Oncology, Department of Obstetrics and Gynecology, Madison, WI, United States

**Keywords:** Melanoma, Metastatic melanoma, Melanoma in pregnancy, Melanoma in ovary

## Abstract

Metastatic melanoma to the ovary is an uncommon presentation. We report a case of metastatic melanoma to the ovary that presented as a growing left adnexal mass during pregnancy and was thought to be benign by imaging and frozen section pathology. Here we discuss the challenges in radiologic and pathologic diagnosis, as well as considerations for the mother and newborn.

## Highlights

• Metastatic melanoma to the ovary is uncommon and can occur years after initial diagnosis.
• Ovarian metastatic melanoma can mimic various benign lesions on imaging and clinical history is key.
• If any suspicion in pregnancy, placenta should be evaluated due to possibility of transplacental transmission.

## 1 Introduction

Cancer is diagnosed in ∼1 per 1,000 pregnancies and the most common cancers are breast, cervical, hematologic malignancies and melanoma (Still and Brennecke, 2017). The incidence of melanoma has been increasing globally with 1/3 of all diagnosis in women occurring in the childbearing age. Transplacental transmission has also been reported in certain malignancies like melanoma, leukemia, lymphoma, lung and breast cancer with melanoma being the most common. Unfortunately, the effect of pregnancy on melanoma continues to be debated and diagnosis based on imaging alone remains difficult. Here, we discuss a case of metastatic melanoma to the ovary during pregnancy presenting as a rapidly enlarging left adnexal mass initially favored to be a hemorrhagic cyst in a patient previously diagnosed with stage IIIA melanoma 10 years prior.

## 2 Case description

A 33yo G4P2022 with history of stage IIIA melanoma was referred to OB/GYN at 25wk gestation for an enlarging left adnexal mass. Her pregnancy was otherwise complicated by gestational diabetes, group B streptococcus and history of depression. She was diagnosed with stage IIIA (stage pT3aN1a) melanoma at age 23 following wide local excision of a lesion on her posterior left shoulder and left axillary lymph node biopsy showed microscopic focus of <1 mm disease in 1/5 nodes. She only completed 1 month of Alfa interferon therapy due to side effects. Her last surveillance visit was two years ago with no evidence of disease. Family history was notable for father with multiple myeloma, prostate cancer and thyroid cancer all diagnosed in his late 60 s, paternal aunt with malignant melanoma in her 20 s, breast cancer in her 40 s and ovarian cancer versus cervical cancer in her 20 s, paternal grandfather with malignant melanoma diagnosed in his 30 s, and mother with type 2 myotonic dystrophy. The patient’s father carries pathogenic variant in CHEK2 gene. Her paternal aunt declined genetic testing. The patient shares her mother’s pathogenic repeat expansion associated with type 2 myotonic dystrophy.

**Fig. 1:**
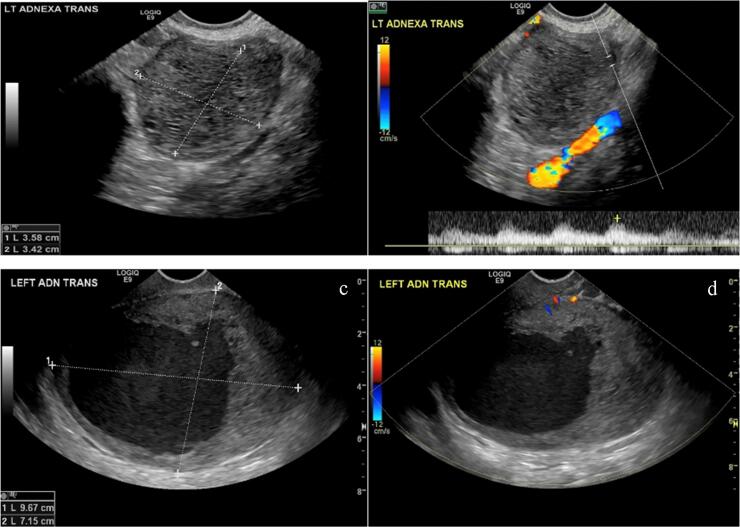
Grayscale and Doppler ultrasound images images of cystic left ovarian mass: (a and b) 12 wk 5 d exam reveals complex cyst with fairly uniform low level internal echoes and no internal blood flow, (c and d) 23 Weeks 4 day follow up exam reveals enlargement of this cystic lesion although portions are more simple appearing fluid. Note difficulty distinguishing walls of the cyst from surround ovarian stroma.

**Fig. 2:**
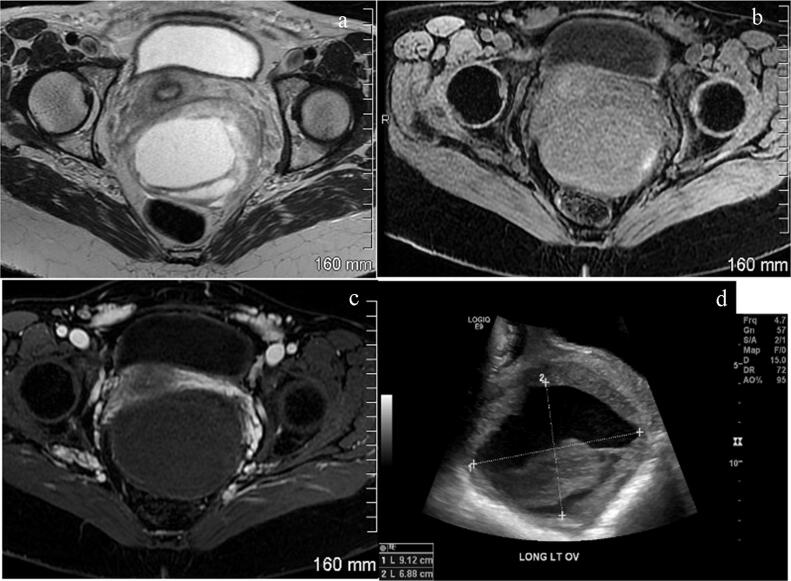
At 27 weeks gestation (a) T2-weighted MRI, (b) T1-weighted MRI, (c) T1-weighted MRI with contrast, demonstrate cystic mass with single septation and intrinsically T1 hyperintense fluid but no solid enhancing internal components following contrast, (d) At 34 week 3 day grayscale ultrasound image show mild continued growth of cystic lesion with solid appearing debris without internal blood flow on Doppler (not shown).

Review of transvaginal ultrasound 3 months prior to her last menstrual period showed no adnexal masses. At her 12w5d ultrasound, she had an enlarged left ovary measuring 5.0 × 4.3 × 3.7 cm and a complex cystic lesion with uniform low-level internal echoes measuring 4.1 × 3.6 × 3.4 cm replacing most of the ovary. No internal blood flow was present within this cystic lesion which was favored to be a hemorrhagic cyst filled with blood clot (Fig. 1). The mass was followed throughout her pregnancy with progressive growth to 11.9 × 9.4 × 11.7 cm by 34wk gestation (Fig. 2). Due to continued growth, MRI was obtained at 27wk gestation, confirming cystic nature of lesion with a single septation but no enhancing components (Fig. 2). The fluid demonstrated intrinsic T1-hyperintensity suggestive of proteinaceous or hemorrhagic fluid. Aside from continued growth, the mass was simple appearing with no imaging features concerning for malignancy, such as discernable thick walls, septations or nodular enhancing components. MRI characteristics favored benign process such as atypical hemorrhagic cyst, decidualized endometrioma, or less likely neoplasm.

Due to the enlarging mass and concern for outlet obstruction, she underwent scheduled cesarean delivery with left salpingo-oophorectomy. Although imaging had consistently favored a benign mass, the possibility of malignancy was discussed with gynecology oncology and decision was made for intraoperative frozen section and additional staging procedure as necessary.

She delivered a 2,630 g female infant with Apgars of 2, 6 and 9 at 38w5d. Intraoperative findings were notable for a 15 cm smooth, mostly fluid filled left ovarian mass with superficial vascularity, internal fat and no obvious normal ovarian tissue. There were no adhesions between the mass and the rest of the pelvis. The abdomen was explored with no palpable masses or nodularity in the pelvis or upper abdomen. Left salpingo-oophorectomy was performed with frozen section favoring decidualized endometrioma or hemorrhagic cyst, and thus, the placenta was not sent to pathology. Final pathology returned as metastatic melanoma.

**Fig. 3:**
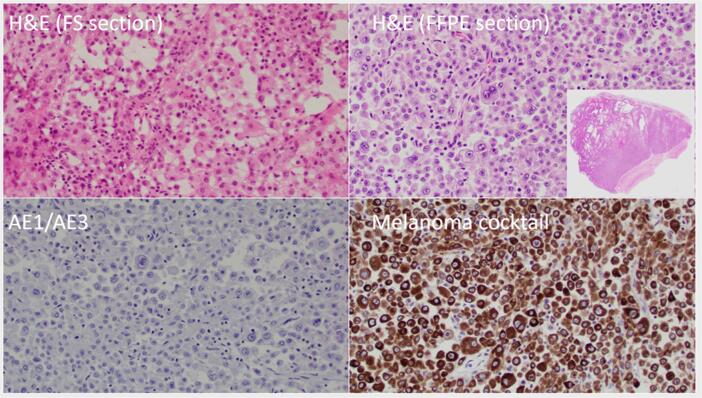
Large dyshesive epithelioid eosinophilic cells portray prominent nucleoli, no mitosis, bi-nucleation on frozen (FS) and permanent (FFPE) sections, and a prominent macro follicular architecture (FFPE inset). Immunohistochemical stains for cytokeratin (AE1/AE3) is negative and melanoma cocktail (HMB-45 and Melan A) is positive.

Pathology findings were notable for an intact ovary with smooth outer surface and filled with hemorrhagic fluid and necrotic debris without a discrete solid component. Representative section at the time of frozen section demonstrated large dyshesive eosinophilic cells with prominent nucleoli, rare to no mitosis, reminiscent of a pregnancy luteoma. The malignant nature of the cells became apparent on examination of additional tissue sections on the formalin fixed paraffin embedded tissue. In addition to the large dyshesive eosinophilic cells with prominent nucleoli, few mitosis, bi-nucleation, marked necrosis, and a prominent macro follicular architecture raised the possibilities of a juvenile granulosa cell tumor, a large-cell variant of small cell carcinoma of the ovary – hypercalcemic type, and metastatic melanoma. Dysgerminoma and undifferentiated ovarian carcinoma were also in the consideration. Based on morphology and patient’s history of melanoma, a limited panel of immunohistochemical stains were performed. The tumor cells were negative for cytokeratin AE1/AE3 and positive for a melanoma cocktail containing HMB-45 and Melan A (Fig. 3). Comparison with the prior pathology was morphologically consistent with metastatic disease. In addition, the tumor demonstrated a BRAFV600E mutation, also specific for metastatic melanoma in the given clinical setting.

Since this diagnosis, patient was referred back to her oncologist and subsequent PET/CT showed involvement of retroperitoneal and left inguinal lymph nodes. Our melanoma tumor board recommended immunotherapy with ipilimumab/Nivolumab every 3 weeks for 4 cycles followed by Nivolumab maintenance therapy with goal of slowing progression. She obtained a second opinion, also recommending immunotherapy or targeted therapy with BRAF/MEK inhibitor given her tumor’s BRAFV600E mutation. The patient is currently receiving ipilimumab/Nivolumab with stable disease.

As for the newborn, pediatric oncology was contacted following maternal diagnosis. The infant was seen by pediatric oncology at 1 week of life with exam notable for a 1 cm lesion on the back. She was then evaluated by dermatology with the lesion described as a 7 mm well-demarcated, symmetric flat nevus with monomorphic brown pigmentation. On dermoscopy, there was a monotonous globular pigment network, consistent with congenital melanocytic nevus, and not concerning for melanoma. As congenital melanoma can be cutaneous or visceral, imaging was also done to screen for visceral lesions. To avoid radiation, the infant has been followed with chest x-ray and abdominal ultrasound every 3 months. Thus far, imaging has remained negative and no new skin lesions have appeared. The infant is closely followed by family medicine, pediatric oncology, radiology, and pediatric dermatology, and currently remains healthy at 5 months of age.

## 3 Discussion

The time interval between diagnosis of primary melanoma and ovarian metastasis has ranged from months up to 18 years. About one third of melanomas in women occur during childbearing age and in Australia, it is the most common malignancy during pregnancy (Still and Brennecke, 2017). In a recent report of 60 patients diagnosed with melanoma during pregnancy, 50% had advanced stage disease with 25% having recurrent disease (Haan et al., 2017). One hypothesis suggest that women of reproductive age may be more susceptible due to higher blood flow to the premenopausal ovary, which may explain the rapid growth of the mass in our patient with increased blood flow in the utero-ovarian vessels with pregnancy. Another recent study suggested that pregnancy-associated plasma protein-A (PAPP-A) may also affect melanoma progression (Still and Brennecke, 2017).

Unfortunately, as with our case, ovarian metastatic melanoma can be a diagnostic challenge on imaging as it can mimic various ovarian entities from benign lesions to primary ovarian malignancy. Most of the reported ovarian metastases have been diagnosed following surgical resection. On imaging, ovarian metastases from most tumor types are often bilateral, solid and strongly enhancing. However, cystic and necrotic areas are common, such that the tumors may be predominantly cystic (as in our case) and resemble primary ovarian cancer. Ultrasound and CT are typically unable to characterize these lesions, but MRI could raise suspicion if there is hyperintense signal on T1 weighted images related to the amount of melanin in the lesion but only one third of the lesions will have this characteristic. The cystic lesion in our case demonstrated T1-hyperintense fluid, but this finding in of itself is non-specific and can be seen with hemorrhagic cyst or endometrioma. Without clinical context of metastatic melanoma, prospective diagnosis remains difficult.

Furthermore, malignant melanoma involving the ovary is uncommon and is often metastatic in origin from a cutaneous site (Fitzgibbons et al., 1987, Young and Scully, 1991, Gupta et al., 2004). Primary ovarian melanoma has also been reported, typically arising in the wall of a mature cystic teratoma, sometimes accompanied by a junctional activity beneath the squamous lining of the cyst (McCluggage et al., 2006). In cases of a pure ovarian melanoma, a careful evaluation for an occult melanoma or the possibility of a regressed cutaneous melanoma should be considered. Clues to metastatic disease include bilateral involvement, involvement in the form of multiple nodules, and smaller (<10 cm) size (Young and Scully, 1991). However, unilateral presentation was the predominant pattern described in the largest series of cases published in the literature akin to our case (Gupta et al., 2004).

Metastatic melanoma may closely resemble a lipid-poor steroid cell tumor, and in a pregnant woman, a pregnancy luteoma. The presence of follicle-like spaces in metastatic melanoma also mimics juvenile granulosa cell tumor, and small cell carcinoma of the ovary, hypercalcemic type. Additional patterns seen in metastatic melanoma involving the ovary include nodular, diffuse, nodular and diffuse, nested and pseudomyxoid areas with a cord-like pattern (Gupta et al., 2004). The varied appearances that metastatic melanoma assumes pose diagnostic difficulties in the ovary, especially in the absence of a previous diagnosis or history. Therefore, clinical history is crucial in the diagnosis. As is often the case, a remote history of cutaneous melanoma is often not considered relevant by the patient or known by the clinician at the time of imaging or during frozen section evaluation. Had her history of melanoma been better communicated with radiology, metastatic melanoma may have been considered which could have expedited surgical intervention. Additionally, although transplacental metastases to the fetus are rare, when it does occur, melanoma metastasis is the most common, accounting for 30% of placental metastases (Potter and Schoeneman, 1970, Brodsky et al., 1965, Alexander et al., 2003). Placental metastasis may also be underestimated because the placenta may not be examined adequately if it appears grossly normal. Unfortunately, the placenta was not evaluated after the delivery of the baby due to benign frozen pathology and is another limitation in this case report as we do not know whether the placenta was involved by melanoma and therefore, the risk to the baby.

Transplacental transmission of melanoma has been recognized, but there are relatively few documented case reports (Holland, 1949). Melanoma can present in infants and young children de novo as well, and the majority of cases are de novo, rather than via transplacental origin (Richardson et al., 2002). Congenital melanoma can also present as cutaneous skin lesions or visceral lesions (Haan et al., 2017). In one case report, it presented as lesions in the CNS (Trumble et al., 2005). Given the high rates of infant involvement, if the placenta is involved, strict monitoring of the infant is warranted, though there is no accepted monitoring plan for these infants. Some have used skin exams, abdominal ultrasounds, or screening for melanogens in the urine (Baergen et al., 1997, Russell and Laverty, 1977, Anderson et al., 1989).

For cases where the mother has known melanoma during pregnancy, the risk for transmission appears to be highest if the placenta is involved. It has been shown that there is approximately a 25% chance of vertical transmission of melanoma if the placenta is positive for disease (Alexander et al., 2003, Baergen et al., 1997). Thus we recommend evaluation of the placenta for patients with known or suspected melanoma at the time of birth. In the case of this mother and infant, the placenta was unfortunately not evaluated. Given that the placental status was unknown, we still opted for a conservative approach with frequent monitoring of the infant. Monitoring is accomplished in this case with skin exams by dermatology, and every 3 month chest x-ray and abdominal ultrasound to avoid radiation, as well as clinical exam by pediatric oncology.

Given these antenatal diagnostic challenges and important considerations, ovarian melanoma should be suspected in any patient with history of malignant melanoma who presents with an ovarian mass without benign cystic teratomas, even if the remission period is long. Finally, interdisciplinary care is also important when treating a pregnant patient with suspected melanoma. Our patient and her newborn were referred to oncology and pediatric oncology and dermatology and are both receiving appropriate treatment and surveillance.

## Author Contributions

Huang DS, MD: Involved in patient’s obstetric care and surgery. Composed majority of the manuscript and helped oversee the manuscript.

Hegeman RB, MD: Patient’s primary oncologist. Helped oversee the manuscript and assisted with the oncology sections of the manuscript.

Roy M, MD: Pathologist involved in patient’s care. Helped with the pathology section of the manuscript and provided the pathology figures.

Prout TM, MD: Radiologist involved in patient’s care. Helped review all of patient’s imaging during her pregnancy and assisted in writing the radiology sections of the manuscript and providing radiology figures.

Swartz K, MD: Involved in patient care. Participated in the write up of the manuscript.

Olsen M, MD: Pediatric oncology fellow in patient’s newborn’s care. Assisted with the pediatric sections of the manuscripts.

Rose SL, MD: Involved in patient’s initial gynecologic oncology consult and surgery. Helped oversee the manuscript.

## Declaration of Competing Interest

The authors declare that they have no known competing financial interests or personal relationships that could have appeared to influence the work reported in this paper.
